# Making Sense of Action Bias in Higher Education: Pedagogical Insights on Critical Thinking

**DOI:** 10.3390/bs15101372

**Published:** 2025-10-08

**Authors:** Faith Jeremiah, Robert Istvan Radics

**Affiliations:** Department of Global Value Chains and Trade, Faculty of Agribusiness and Commerce, Lincoln University, Lincoln 7647, New Zealand; robert.radics@lincoln.ac.nz

**Keywords:** action bias, critical thinking, higher education, learning and teaching

## Abstract

Action bias, the cognitive tendency to favor action over inaction regardless of its necessity, has been extensively studied across domains such as behavioral economics, organizational behavior, and policy development. However, its manifestation in educational contexts remains critically underexplored. In the digital age, with an abundance of both factual and misleading information, the persistence of action bias within education jeopardizes the cultivation of initial critical thinking capable of addressing multifaceted global challenges. The analysis indicates how institutional norms may foster a performative academic identity that conflates speed and compliance with intellectual competence. Through workshops conducted with university students ranging from undergraduate to PhD levels, participants were tasked with solving a practical yet ambiguous problem to highlight potential cognitive differences across educational stages. Despite prior training in critical thinking, participants consistently defaulted to immediate ideation, bypassing fundamental inquiries into the problem’s legitimacy or broader implications. Using a sensemaking approach, this study demonstrates that reflexive actions are not interpreted as merely cognitive shortcuts but behaviors shaped by educational systems prioritizing visible outputs over critical inquiry. The findings reveal how institutional norms foster a performative academic identity, conflating speed and compliance with intellectual competence. This research challenges traditional pedagogical models, advocating for educational reforms that emphasize assessing the *process of learning*. By situating action bias within the broader framework of active learning, this study offers actionable insights for educators, policy makers and researchers to foster critical innovative thinking, essential in an increasingly digital future.

## 1. Introduction

“*[…] it’s easier to just accept everything we are told or read.”*

(Postgraduate participant, Workshop C)

At its core, education serves as the crucible for intellectual exploration, shaping individuals who can confront the unpredictable challenges of an increasingly complex world. It is here, amidst the interplay of curiosity and critique, that innovation and critical problem-solving emerge, capacities that transform theoretical insights into real-world advancements (Sternberg & Tardif, 2009). Yet, even in environments dedicated to intellectual growth, cognitive processes are not immune to bias. Among the most insidious of these is action bias, the tendency to favor action over inaction, even when action may not be warranted (Patt & Zeckhauser, 2000).

Action bias offers the seductive illusion of decisiveness, a quality often celebrated in educational and professional settings. However, this inclination frequently circumvents critical reflection, leading to hasty, suboptimal outcomes (Ariely, 2008; Kahneman, 2011). The ripple effects of this bias have been extensively documented in fields such as behavioral economics, environmental policy, sports psychology, and organizational behavior, where it is often fueled by psychological pressures like uncertainty avoidance and the need to appear proactive (Bar-Eli et al., 2007; Dykstra et al., 2022). Curiously, while education shapes the very cognitive and decision-making frameworks that underlie these domains, the presence and implications of action bias within educational contexts remain strikingly underexplored.

This gap is particularly disquieting considering the modern world’s demand for intellectual agility. As global challenges grow more intricate and multifaceted, the ability to balance decisiveness with reflective inquiry becomes ever more critical. This tension echoes broader evidence that innovation capacity in universities stalls when organizational learning systems overlook cognitive barriers (Li et al., 2023). How then, does an education system intended to cultivate thoughtful, adaptive learners potentially perpetuate cognitive shortcuts that undermine these goals? To investigate, this study examines the manifestation of action bias within higher education, focusing on how it influences problem-solving behaviors among university students.

Through a series of exploratory workshops, participants were presented with an ostensibly simple challenge: retrieving items from high supermarket shelves. Despite prior instruction in critical thinking, students repeatedly defaulted to generating immediate solutions, bypassing foundational inquiries into the problem’s legitimacy or broader implications. This dynamic reveals more than a heuristic; it signifies a deeper, systemic issue in which educational norms prioritize rapid output over thoughtful analysis and deliberate reflection (Daff et al., 2024; Philp-Clark & Grieshaber, 2024).

Using a sensemaking approach, this research challenges the assumption that action inherently equates to progress. It situates action bias within the cultural and institutional fabric of educational settings, where performative academic identities, marked by compliance and efficiency, seemingly take precedence over intellectual resilience and inquiry. In this study, we use the term performative academic identity to denote a mode of self-presentation in which quick, compliant task performance is treated as a proxy for competence, often sidelining inquiry and critical reflection. By highlighting these patterns, this study advocates for a reimagining of pedagogical approaches that prioritize inquiry-driven problem-solving and critical reflection, essential competencies in this digital era.

Although action bias has been widely studied, little is known about how it varies across stages of higher education or how individuals interpret and rationalize their actions in creative problem-solving. To address these gaps, this exploratory qualitative multi-group workshop study is guided by the following research questions:

1. How does action bias manifest across different educational levels, from first-year university students to postgraduates, during creative problem-solving?
2. How do participants interpret and make sense of their experiences related to action bias?

These questions frame the exploratory workshop design and guide the grounded theory–sensemaking analysis that follows.

## 2. Literature Review

### 2.1. Systematic Review Procedures

**Figure 1:**
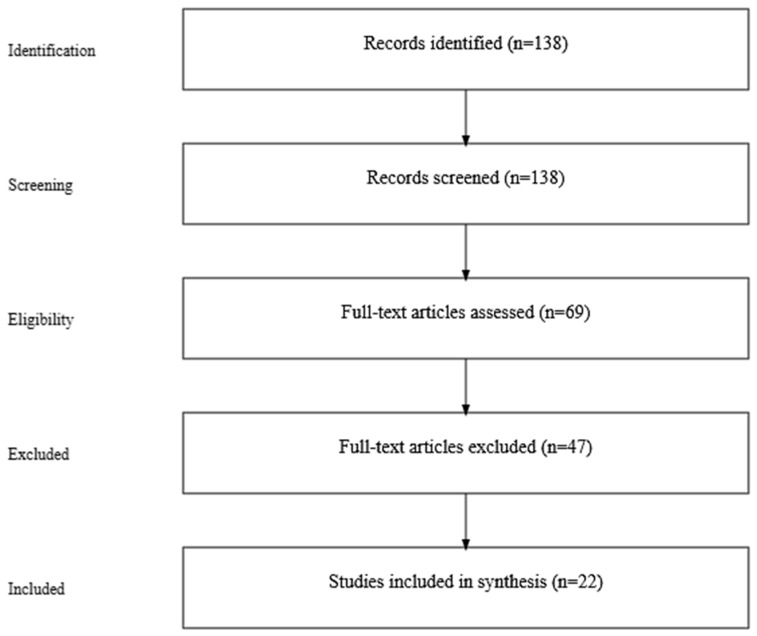
PRISMA flow diagram.

Searches were conducted in Scopus and Google Scholar between August 2024 and November 2024 using the terms ‘action bias’, ‘action bias AND education’, and ‘action bias AND innovation’. Records were deduplicated, then screened by title and abstract against inclusion criteria: peer-reviewed sources in English, empirical or theoretical relevance to action bias, and direct applicability to decision-making or learning contexts. Exclusion criteria included non-English sources, ‘single action bias’ in climate-change literature not addressing general action bias, inaccessible full texts, and items only tangentially referencing action bias. Full texts were then assessed for eligibility. Screening was conducted by the first author; no inter-rater reliability statistics are reported. A PRISMA flow diagram (Figure 1) and a supplementary table of inclusion and exclusion reasons (Table 1) document each stage.

### 2.2. Scope and Generalizability

This study does not assume universal applicability across cultural contexts. Much behavioral science evidence remains disproportionately WEIRD (Western, Educated, Industrialized, Rich, Democratic), and the English-language search likely reinforced this overrepresentation. Accordingly, the claims are interpreted as context-bound, with a call for cross-cultural replications (e.g., Begeny et al., 2018, 2021; Thalmayer et al., 2021; van de Vijver, 2013).

### 2.3. Review Objectives and Approach

A systematic review was undertaken to critically examine the existing body of research on action bias, with two primary objectives: to map previously studied domains and to identify key gaps in the literature. The methodology followed established protocols for conducting systematic reviews (Radics et al., 2015), ensuring both rigor and transparency throughout the process.

The initial search was conducted using the Scopus database with the keyword “action bias,” which yielded 95 results. Titles and abstracts were screened for relevance and language, resulting in 26 articles that met the preliminary inclusion criteria. Studies focusing on “single action bias,” a concept prominent in climate change literature but beyond the scope of this study, were excluded. After further evaluation for accessibility and direct relevance to the research questions, 10 articles were selected for in-depth analysis.

To broaden the contextual reach and reduce redundancy, a secondary search was conducted via Google Scholar using the keywords “action bias AND education” and “action bias AND innovation.” The first query identified 28 education-related articles, nine of which met the inclusion criteria. The second query yielded 15 results on innovation, with three ultimately retained. Duplicate and tangentially related sources were excluded from the final pool.

Where necessary, articles were accessed through interlibrary loan services spanning national and regional academic networks. This ensured a comprehensive and diverse source base, free from institutional bias. A summary of the search process and selection criteria is presented in Table 2. While Figure 1 presents the overall PRISMA flow, Table 2 complements it by detailing the search queries. It shows the databases and keywords used, initial yields, screening decisions, and final inclusion counts, information not captured in the aggregate diagram.

### 2.4. Action Bias in the Literature

This literature review highlights that action bias has attracted widespread scholarly attention across diverse disciplines, reflecting its significant influence on decision-making. Researchers have explored action bias in environmental and consumer choices (Patt & Zeckhauser, 2000); natural resource management policy (Iftekhar & Pannell, 2015); law and policy development (Sunstein & Zeckhauser, 2011; Reason, 2009); sports (Bar-Eli et al., 2007; Lamiraud & Vranceanu, 2018); cybersecurity (Dykstra et al., 2022); leadership (Noel & Erskine, 2013; Paukku & Välikangas, 2021); financial markets and behavioral economics (Shiller, 2000); health (Iserson, 2024); and entrepreneurship (Noel & Erskine, 2013).

The breadth of research highlights the multifaceted nature of action bias and its capacity to shape outcomes across domains, especially when paired with proactive behaviour and critical thinking. Patt and Zeckhauser (2000), for instance, note that individuals often act on personal beliefs or perceived responsibility, even when such actions lack rational justification. This tendency also affects natural resource management, where policymakers, eager to appear proactive, may implement visible but suboptimal measures (Iftekhar & Pannell, 2015). However, while action bias clearly emerges across fields, its expressions vary by context. In law and policy, the drive to appear decisive often leads to short-term fixes that undermine long-term solutions (Sunstein & Zeckhauser, 2011; Carland et al., 2019). In sport, competitive pressure prompts impulsive choices at the expense of better strategies (Bar-Eli et al., 2007; Lamiraud & Vranceanu, 2018). Similarly, in cybersecurity, urgency can override critical risk assessment (Dykstra et al., 2022).

Action bias also manifests in leadership, where the urge to appear decisive can lead to impulsive choices misaligned with organizational goals (Noel & Erskine, 2013; Paukku & Välikangas, 2021). In financial markets, emotional reactions to volatility often override rational judgment, while in healthcare, urgency may drive interventions unsupported by strong evidence (Iserson, 2024). Entrepreneurship, too, reflects this tension—rapid pursuit of opportunity can spark innovation but heightens exposure to risk and failure (Noel & Erskine, 2013). Collectively, these examples underscore how action bias is contextually shaped by domain-specific norms, pressures, and expectations.

### 2.5. Unpacking Action Bias

Action is often perceived as inherently valuable, particularly in comparison to inaction. This bias toward action reflects societal and psychological norms that equate decisiveness with competence and productivity, even in situations where restraint or deliberation might yield better outcomes (Dykstra et al., 2022). Carland et al. (2019) underscore this dynamic, noting that action is often viewed favorably regardless of whether its consequences are positive or negative. While prompt action can foster positive outcomes or benefit others, when the compulsion to act overrides logical deliberation (Ormerod et al., 2013), it has a tendency to produce suboptimal or unwarranted outcomes (Patt & Zeckhauser, 2000).

From a cognitive perspective, action bias is rooted in the fast, intuitive processes of System 1 thinking described by Kahneman (2011). System 1 operates as a mental shortcut that prioritizes rapid decision-making, often at the expense of the analytical rigor characteristic of System 2 thinking. This tendency is particularly pronounced in situations of uncertainty, where individuals act pre-emptively to regain a sense of control, even when the actions themselves lack utility or coherence (Dykstra et al., 2022). For example, in crises or ambiguous problem contexts, the cognitive discomfort of inaction often compels individuals to seek immediate solutions, irrespective of their effectiveness.

Interestingly, action bias emerges early in human development. Forbes and Farrar (1993) found that children as young as 24 months display a preference for action in their linguistic behaviors, suggesting it may be deeply embedded in human cognition. While heuristics like action bias can streamline decisions and improve efficiency, they are also error-prone. As Reason (2009) notes, overreliance on such shortcuts can constrain creativity, stifle innovation, and hinder effective decision-making, especially in situations demanding critical reflection or novel solutions.

#### 2.5.1. Emotional and Contextual Influences

The role of emotion further amplifies action bias. Fear and anxiety, which are common responses to uncertain or high-stakes situations, amplify the urgency to act, often bypassing critical evaluation of alternatives (Sunstein & Zeckhauser, 2011). This is particularly evident in what Sunstein and Zeckhauser term fearsome risks, where emotional arousal leads to overestimations of threat and disproportionate responses. Such reactions are not limited to individuals; they extend to organizations and governments, which may overreact to perceived dangers, failing to distinguish between high- and low-risk scenarios.

The organizational and societal consequences of action bias are significant. Research demonstrates that premature decisions, driven by the need to act, often bypass critical stages of evaluation, resulting in missed opportunities and overlooked alternatives (Dykstra et al., 2022). This dynamic is particularly problematic in creative problem-solving contexts, where innovation depends on the capacity to question assumptions and explore diverse options (Paz-Baruch et al., 2025). Institutional and cultural norms that privilege productivity and visible outcomes may further entrench this bias, reinforcing a preference for swift resolution over reflective inquiry (Leibovitch et al., 2025; Yulin & Danso, 2025).

This cultural embedding of action bias is evident across organizational domains. For example, Decker et al. (2022) found that wildlife management organizations often prioritized action over careful planning, influenced by historical practices and institutional incentives. However, these reactive strategies frequently produced suboptimal results. Similarly, in social entrepreneurship, action bias has been identified as a factor in a reductionist cycle, where premature solution generation obscures the complexity of social problems and inhibits effective problem-solving (Chipeta et al., 2022). Within such contexts, the normalization of action bias is increasingly recognized as a systemic concern (Patt & Zeckhauser, 2000; Dykstra et al., 2022).

#### 2.5.2. Balancing Action and Reflection

Though often problematic, action bias is not inherently detrimental. In emergencies, it can expedite decisions and reduce hesitation (Cioffi, 2025). However, when left unchecked, especially in contexts requiring deliberation, it can undermine reflective thinking. To mitigate this, we posit the need to cultivate educational environments that balance decisiveness with reflection, encouraging System 2 thinking throughout students’ learning journeys. While this heuristic may enhance responsiveness, its overuse in complex or novel contexts, where deeper analysis is required, can lead to suboptimal outcomes (Shye & Viale, 2025). In such cases, overreliance on cognitive shortcuts may constrain innovation and hinder exploration, analysis, and creative problem-solving (Borowa et al., 2025).

### 2.6. Educational Norms and Cognitive Processing

Deep, reflective thinking is arguably one of the most challenging yet essential skills to foster in education (Sudirman et al., 2024). It demands sustained effort to critically engage with ideas, evaluate assumptions, and synthesize insights—practices often misaligned with the structural and cultural norms of contemporary education (Fynes-Clinton et al., 2024). Although research shows that reflective thinking enhances academic performance, decision-making, and problem-solving (Sudirman et al., 2024), it remains undervalued in systems that prioritize visible outputs over intellectual depth (Halpern, 1998, 2013). The limited academic focus on action bias within educational contexts further signals how this crucial dimension of learning continues to be overlooked. Such norms mirror the capability-drain universities face when knowledge is not translated into innovation processes (Lytras et al., 2022).

Recent research in reinforcement learning offers valuable insight: individuals who struggle with learning often exhibit strong cognitive biases, yet even high-achieving learners show similar patterns (Colas et al., 2024). This suggests that cognitive biases are not confined to deficits in ability but are embedded within the learning process itself, complicating efforts to foster meaningful educational outcomes (Patt & Zeckhauser, 2000; Dykstra et al., 2022). More significantly, this points to action bias not merely as an individual tendency, but as a culturally reinforced norm within education. The pressing challenge is to shift these ingrained mindsets and deliberately cultivate environments that support reflective thinking and proactive inquiry.

### 2.7. Higher Education and Critical Thinking

Critical thinking, encompassing reflective judgment, logical analysis, and self-regulation, remains a cornerstone of higher education (Halpern, 1998, 2013). While early frameworks focused on formal reasoning and argumentation (Bailin et al., 1999), contemporary perspectives have expanded to include metacognition, social critique, and discipline-specific practices (Brookfield, 2013; Tsui, 2002). Debates persist over whether critical thinking is primarily a domain-general competency transferable across various fields or more context-dependent, requiring discipline-specific knowledge and practices (Bailin et al., 1999; Halpern, 2013; Liu et al., 2014). Nonetheless, many researchers endorse blended approaches that couple generic thinking strategies with subject-specific applications (Ennis, 2011; Tsui, 2002).

Pedagogical research underscores two dominant models for embedding critical thinking in curricula: stand-alone courses and infusion within disciplinary teaching (Abrami et al., 2015). Stand-alone programs typically focus on logic, argumentation, and metacognitive strategies, aiming to directly develop students’ analytical capacities (Halpern, 1998; Ennis, 2011; Elder & Paul, 2009). Infusion approaches, by contrast, integrate reflective thinking and problem-solving into standard academic subjects, employing methods such as problem-based learning, case studies, and Socratic dialogue to foster deeper cognitive engagement and application of reflective thinking (Gijbels et al., 2005; Liu et al., 2014).

Furthermore, a subset of literature informed by critical pedagogy underscores how sociopolitical awareness is integral to critical thinking, urging educators to interrogate power structures, cultural norms, and fostering students’ ability to think critically about both academic content and societal dynamics content (Freire, 2020; Brookfield, 2013).

While the literature converges on the centrality of critical thinking in higher education, it diverges on optimal pedagogical and assessment strategies (Bailin et al., 1999; Elder & Paul, 2009). Thus, the assessment of critical thinking presents unique challenges. Standardized instruments like the California Critical Thinking Skills Test provide valuable benchmarks but may fail to capture the nuanced, context-dependent reasoning processes that characterize discipline-specific expertise (Ennis, 2011; Shavelson, 2010). In contrast, authentic, discipline-embedded evaluations (e.g., portfolios, reflective journals, and performance tasks) can capture richer insights but often lack standardization and scalability (Nicol & Macfarlane-Dick, 2006).

Even so, the emerging consensus highlights the importance of contextualized, reflective instruction that combines universal cognitive processes with discipline-specific content, supported by robust, formative evaluation mechanisms (Brookfield, 2013; Halpern, 2013; Abrami et al., 2015). Perhaps this is why increasingly scholars advocate mixed-method assessments, such as merging standardized testing with qualitative, discipline-specific tasks, to produce a more comprehensive picture of students’ critical thinking abilities (Abrami et al., 2015; Shavelson, 2010).

### 2.8. The Gap

Despite a rich body of research on action bias, its manifestation within education appears largely unexamined. Existing studies focus primarily on the role of action bias in immediate decision-making contexts, such as crisis management or policy implementation, leaving the long-term influence of educational structures and norms on cognitive tendencies underexplored. This gap is particularly significant given education’s role in shaping not only knowledge acquisition but also the lifelong cognitive habits that underpin decision-making.

While existing research shows that external pressures, such as deadlines or risk aversion, reinforce action bias, little attention has been given to how these dynamics manifest in academic settings. Task-oriented pedagogies that emphasize speed and measurable outcomes may risk sidelining reflective thinking (Sudirman et al., 2024; Schürmann et al., 2025). This raises the question of to what degree such norms might limit students’ capacity to navigate complex, real-world challenges. Moreover, the developmental path of action bias is poorly understood. Few studies have investigated how cognitive tendencies shift from early education through higher education into professional practice. This absence of longitudinal research limits understanding of how academic environments shape long-term decision-making patterns, leaving a significant gap in both theory and application.

Accordingly, we investigate action bias in higher education using an exploratory, multi-group workshop design and a grounded theory–sensemaking analysis to examine how it manifests across levels and how students make sense of it.

## 3. Methodology

An exploratory multi-group workshop design was employed to elicit real-time problem-solving and subsequent sensemaking (Boud et al., 2014; Kolb, 2014). This approach surfaces interactional dynamics and cognition under ambiguity but offers depth at the expense of broad ecological generalizability and can be sensitive to facilitation and group effects (Charmaz, 2006; Corbin & Strauss, 2014). To mitigate these risks, prompts were standardized and immediate reflections were captured to enhance interpretive authenticity (Gioia et al., 2013; Sandberg & Tsoukas, 2015). The design aligns with inductive qualitative approaches (Charmaz, 2006; Corbin & Strauss, 2014) and builds on interactive, workshop-based methods widely used in higher education (Boud et al., 2014). For precedents in inductive analysis and interpretive rigor, scholars have shown that such approaches capture emergent patterns of meaning-making (Gioia et al., 2013; Sandberg & Tsoukas, 2015). Analytically, grounded theory and sensemaking principles were integrated and refined iteratively to allow patterns and themes to emerge without imposing pre-existing theoretical constructs (Charmaz, 2006; Corbin & Strauss, 2014), a strategy well-suited to examining context-dependent, cognitively embedded phenomena such as action bias.

### 3.1. Data Collection

The motivation behind this exploratory study stemmed from an insightful classroom experiment to foster innovative and creative problem-solving among undergraduate students. The lecturer introduced a personal challenge related to her difficulty reaching high supermarket shelves. Despite students being taught the importance of being customer-centric and understanding the customer’s problem in previous classes, these students demonstrated a common cognitive predisposition: the impulse to ideate and innovate without fully grasping the problem’s scope or significance. This experiment inspired the authors to replicate it with university students across the campus.

Students were recruited via posters displayed across the university and were selected in order of application until each group was filled. This study was open to students of all ethnic backgrounds and academic disciplines, except for former students of the lead author, to avoid potential conflicts of interest. The lead author facilitated all sessions. This role may introduce authority-related demand characteristics; this issue is acknowledged in this study’s limitations.

The exploratory study involved three workshop groups, each with six students (N = 18 total). Group A comprised first-year students, Group B second- and third-year students, and Group C postgraduate students. Participant demographics are in Table 3. In addition, three supplementary workshops were conducted with only one individual from each university level to explore if group dynamics influenced student behaviors during the innovative problem-solving sessions. The findings showed almost no differences between the individuals and the groups; therefore, the decision was made to focus solely on the groups. These exploratory checks suggested similar patterns across levels; however, with only one workshop per level, the possibility of group-specific dynamics cannot be excluded. This limitation is noted in the Limitations.

Each workshop began in a relaxed atmosphere where students enjoyed a free lunch, drinks, and snacks. The room was filled with lively conversation, intentionally designed to break the ice, build familiarity, and create a comfortable space that encouraged collaboration and a free flow of ideas. Once everyone had finished eating, the lead author announced the start of the session. It is important to note that manipulation checks of psychological safety or collaboration climate were not collected, and this omission is acknowledged as a methodological limitation.

To begin, students were briefly coached on the importance of critical thinking. The lead author then presented a personal challenge: she was too short to reach items on the top supermarket shelf and explained that the issue did not justify using click and collect, as she was typically only picking up a few items. Students were invited to ask questions if they wanted further details. This setup was designed to simulate real-world problem-solving conditions, where information is often incomplete, and challenges are ambiguously defined.

Participants were encouraged to explore innovative solutions. Each group was provided with colored markers, a large whiteboard, pens, paper, sticky notes, and other stationery. All participants, including the lead researcher, sat together around the table.

The lead author facilitated all workshops, which were held on different days and times, each lasting approximately 90 min. With participants’ consent, sessions were recorded in accordance with ethical standards approved by the Human Ethics Committee (HEC). Immediately following each workshop, a relaxed, recorded group discussion was conducted to elicit students’ thought processes and rationale. All sessions were transcribed for analysis. Quotations are attributed as [Group–Participant], for example [A-P3] refers to Group A (first-year), participant 3; [C-P2] refers to Group C (postgraduate), participant 2.

### 3.2. Analysis

Grounded theory principles guided the analytical process, offering a systematic yet adaptive framework for developing theory emergent from the data (Charmaz, 2006). Analysis commenced with open coding to identify initial concepts and latent patterns, which were then refined through axial coding. This second phase enabled articulation of relationships among categories and subcategories, including causal mechanisms, contextual conditions, and consequential dynamics (Corbin & Strauss, 2014).

This study adhered to established criteria for theoretical saturation, ceasing analysis when no new categories or connections emerged (Guest et al., 2006). This ensured analytical depth and completeness while remaining true to the grounded theory ethos of theory construction through iterative immersion in the data. Reflexive memo-writing was used throughout to document decision points and make interpretive influences explicit, providing a transparent record of how meaning was constructed (Charmaz, 2006). Primary coding and memo-writing were conducted by the first author, an entrepreneurship educator whose professional background informed, but also shaped, interpretation. Category structures were reviewed with the second author to test coherence and strengthen credibility. Where relevant, a 20% sample was double-coded to explore category boundaries, with insights from those discussions informing refinements. Formal inter-coder reliability statistics were not calculated given this study’s interpretive orientation. In cases where coding was single-researcher, peer debriefs were used to review decisions and maintain analytic rigor.

The analysis followed an inductive approach, with abductive reasoning used to refine interpretations as patterns developed. Rather than beginning with predefined hypotheses, concepts from the action-bias literature acted as orienting ideas that focused analytic attention without constraining discovery. The resulting theorization is interpretive and situated, offered as a generative framework for further inquiry rather than as confirmatory evidence.

A complementary sensemaking lens added depth by focusing on how participants constructed meaning around their actions and decisions. Operationally, coding and memoing attended to (a) how participants framed the problem, (b) justifications for acting or not acting, and (c) references to social norms or authority. These dimensions guided axial integration while allowing inductive categories to emerge. Grounded theory scaffolded the coding process, whereas sensemaking highlighted the cognitive and sociocultural logics through which behavior was rationalized (Sandberg & Tsoukas, 2015). This dual-method strategy enabled the analysis to interrogate both behavioral patterns and the institutional structures shaping them, addressing broader calls to bridge micro-level cognitive processes with macro-level cultural dynamics (Gioia et al., 2013).

## 4. Findings and Discussion

### 4.1. Action Bias

The findings revealed that across all educational levels, students consistently demonstrated a tendency to engage in immediate problem-solving actions without thoroughly analyzing the problem context, despite being encouraged to ask questions. Initially, participants generated a steady stream of ideas, with ideation continuously active for approximately 45 min. However, as the flow of new ideas diminished, the focus shifted toward reviewing and critiquing their existing suggestions. This phase was marked by expressions of frustration and guilt over the perceived inadequacy of their solutions. Cross-case comparison did not reveal systematic differences by educational level in initial problem framing or in the shift from ideation to self-critique. Where minor variation appeared, postgraduates tended to propose more complex systems, while first-years generated higher idea volume; these tendencies did not alter the core pattern.

A particularly notable feature of the ideation process was the participants’ inclination to generate complex solutions, as one participant explained, [A-P3] “*Well, we’re here to solve this problem, which involves intricate ideas, ya know, the simple ideas seem too simple*.” This excerpt, representative of participants’ collective sensemaking, provided insight into the rationale behind the range of inventive yet impractical ideas proposed, including, [B-P1] “*rotating aisles where you press a button, and the item you want revolves down to a reachable level*,” or [C-P3] “*an app where you select an item and someone brings it to you as you move through the aisles*.” Others suggested structural overhauls like [A-P4] “*making a trolley with a foldable stair lift*” or even reimagining the shopping process as [B-P4] “*a complete automated serviced drive-through type shopping experience*.” These ideas, while demonstrating ingenuity, consistently bypassed considerations of feasibility, usability, and alignment with the given problem.

As the flow of ideas eased, participants began to critically evaluate their own ideation, revealing consistent patterns of sensemaking across the groups. For example, one student remarked to another, [C-P5] “*It feels like we’re using a sledgehammer to crack a nut*,” while another questioned the utility of a proposed grabber tool: [C-P3] “*The grabber tool sounds cool, but are people really going to use it? […] In a rush, I’d probably just try to climb up the shelves*.” These reflective comments highlight a shared recognition of the limitations inherent in their proposed solutions. Interestingly, through these discussions, participants unwittingly constructed their understanding of action bias, articulated through reflections, such as one student’s observation: [B-P6] “*It feels like we were actually solving the problem, just because no one asked if it mattered*.” This realization gradually fostered a collective awareness of their oversight: [A-P3] “*Well, you’re given an assignment or a task, and your brain just goes, ‘Alright, time to fix this,’ instead of thinking, ‘Wait, does this even need fixing?’*”

To challenge the participants’ approach and further examine their sensemaking, the lead author posed a pivotal question: why had no one asked about the actual importance of the problem? She explained that, had they asked, she would have responded by revealing that the issue accounted for just 0.000001% of a problem and was therefore inconsequential. To emphasize this oversight, she erased the whiteboard, highlighting that this single inquiry could have saved the group 45 min of effort. This intervention was deliberately designed to elicit participants’ sensemaking regarding their impulsivity to generate solutions.

### 4.2. Why Did Action Bias Occur

When questioned on their impulsivity to solve, participants’ sensemaking revealed that it was more than a general cognitive heuristic; instead, a more complex behavior reinforced by systemic psychological, physiological and environmental dynamics.

Psychologically, prompt action was explained as a method to avoid the discomfort experienced between thinking and action: [C-P2] “*it’s not right to just think and do nothing*.” This strong cognitive drive to alleviate such tensions was articulated by another, [A-P1] “*I know I needed to at least do something, so I’m glad I started firing out ideas, otherwise its quite awkward*”. This suggests the ‘act of doing’ restored a sense of control (Sunstein & Zeckhauser, 2011) and equilibrium, and even offered psychological gratification from perceived productivity. Thus, the act of solving, whether warranted or not, could be understood as synonymous with competence, as reflected here: [B-P2] “*I didn’t want to look like I wasn’t participating or, worse, like I wasn’t capable.*”

Physiologically, research on irrational decision-making demonstrates how individuals derive physical satisfaction from the act of doing (Harmon-Jones et al., 2024), even when their actions lack substantive value (Campbell et al., 2024, preprint). This phenomenon was evident throughout students’ reflections, for example: [A-P6] “*It feels good—like we’re solving the problem, even if we didn’t actually solve it.*” Another explained, [A-P5] “*I feel good to be actively contributing, it’s got to look good too that we are actively trying rather than just thinking*”.

Participants shared a common explanation of implicit social or hierarchical contracts within the educational environment. The fear of social missteps, such as appearing disengaged or incompetent, fosters a culture of conformity, as shared by this participant, [B-P1] “*If I spoke up and said, ‘Is this really a problem?’ and it turned out that it was a big deal, then I’d look like an idiot for even asking.”* Another explained, [C-P6] “*There’s a subtle pressure to deliver solutions, and the moment you start questioning, it feels like you’re… questioning everything*.” Such widely experienced sentiments underscore how normative pressures in specific environments can reinforce the visible metrics of productivity, a dynamic also observed in organizational contexts where ‘busyness’ is prioritized over thoughtful planning (Decker et al., 2022).

#### 4.2.1. Educational Conditioning

Beyond the psychological, physiological and environmental dimensions, there was the revelation that educational institutions themselves were experienced as the very catalyst for causing action bias. Participants’ sensemaking reveals their interpretations of shared norms that valorize speed and visible outputs (Halpern, 1998, 2013; Sternberg, 2002), for example: [C-P1] “*You’re not thinking about whether the problem matters when you only have a certain amount of time to show you can solve it.*” Another reflected, [A-P3] “*It’s like, at uni we see a problem, and our instinct is to fix it without questioning whether it needs fixing—like, especially in timed exams where you could lose marks.*”

In line with this, Hertwig and Erev (2009) also found that environments driven by external validation intensify output-oriented tendencies, reinforcing the association between visible productivity and intellectual success. This observation suggests a concerning implication: critical inquiry and innovative thinking risk being suppressed, with educational norms seemingly setting the stage for similar dynamics in the workforce (Daenekindt & Huisman, 2020). Freire’s (2020) critique of the banking model of education serves as a prescient warning. By treating students as passive repositories of information, such systems devalue the metacognitive skills essential for contextual evaluation and explorative thinking.

Empirical studies reinforce these theoretical critiques. Recent meta-analyses reveal that students subjected to timed and task-oriented assessments demonstrate reduced reflective capacities, as measured by metacognitive engagement inventories (Gernsbacher et al., 2020). In contrast, longitudinal studies in STEM education demonstrate that students trained in inquiry-based learning outperform their peers in problem-solving tasks requiring contextual adaptability (Hallström & Schönborn, 2019). Similarly, programs that integrate metacognitive checkpoints and require justification of problem relevance have shown measurable improvements in cognitive skills and adaptability (Boud et al., 2014).

#### 4.2.2. Authority Bias and Its Reinforcement of Action Bias

Participants interpreted experiencing authority bias as a product of their perceived systemic conditioning. Authority bias is defined as the tendency to defer to perceived authority figures without critical evaluation (van der Toorn et al., 2011). Part of this was in how they made sense of educators’ expertise and legitimacy, for example, [A-P4] “*If the lecturer brought it up, it must be important… I didn’t even stop to question that. I just trusted that if it was on the table, it was already something that needed fixing*.” This trust seemingly equates instructor-endorsed tasks with significance, as another rationalized: [B-P3] “*It’s like it’s our job to do the task without questioning it, lecturers know more, and they see the relevance even if we can’t.*”

Authority bias has deep roots in the historical evolution of educational systems, many of which were founded on hierarchical models designed to disseminate established knowledge efficiently (Okonofua et al., 2016; van der Toorn et al., 2011). This structure was institutionalized during the Industrial Revolution when education systems were standardized to meet the needs of emerging bureaucratic and industrial workplaces. Such priorities created environments that prioritized compliance, task completion, and hierarchical deference, embedding authority bias as a structural norm (Okonofua et al., 2016).

Yet, perhaps today, this bias is still inadvertently perpetuated through grading systems, standardized assessments, and performance metrics that reward adherence to directives over inquiry, as this participant interpreted: [A-P6] “*We have always, right from kindergarten […] all the way through uni have had to follow orders and do as told without question*”. Research (e.g., Friesen & Osguthorpe, 2018) also indicates that evaluative frameworks normalize experiences that derive from institutional priorities and often inadvertently penalize dissent or unconventional approaches. This was also the shared experience across participants, for example: [B-P5] “*You get the sense that questioning isn’t worth it—it just slows things down and makes you look like you don’t get it*.” This collective sentiment illustrates how authority bias becomes a learned response to environments that valorize speed, compliance, and visible productivity (Gültekin, 2024).

Theoretical perspectives, such as Foucault’s (2012) concept of disciplinary power sheds light on how institutional practices enforce conformity through subtle mechanisms of control, including surveillance and evaluation. These systems continually shaped students’ sensemaking by creating a feedback loop where compliance is rewarded: [B-P6] “*we get rewarded in the form of grades […] or punished in the form of losing marks*”, and critical inquiry is marginalized: [C-P4] “*questioning would be us not knowing our place. You just know not to do it*.” Authority bias, in this context, seems less a cognitive flaw than a rational adaptation to institutional imperatives.

Participants also interpreted instructors as proxies for task relevance, as this participant rationalized: [A-P3] “*They obviously know their stuff. We don’t want to be seen as argumentative or like we are being smart—it’s not worth the risk on our grades*”. This heuristic-like behavior aligns with findings from experiments on obedience (Milgram, 1963), which revealed how perceived authority overrides personal judgment, even in ethically ambiguous scenarios. Again, it seems institutional norms further reinforce this connection. The interplay between authority and action biases thus seems self-reinforcing: students defer to authority-driven tasks and act upon them without interrogating their broader implications, perpetuating a cycle of uncritical engagement.

#### 4.2.3. The Performative Academic Identity

Authority bias fosters what Sternberg (2002) describes as a performative academic identity. Recent research on academic identities demonstrates similar dynamics, showing how institutional norms can frame questioning as disruptive to productivity, making conformity appear safer than critical engagement (Xu & Barrow, 2024). Participants in this study reflected on this tension, with one observing, [C-P6] “*The moment you start questioning, it feels like you’re questioning everything, and that’s not what you’re supposed to do*.” Additionally, another explained, [C-P1] “*like who are we to question? Ya know—they say, and we do. That’s just how it is.”*; another said*,* [A-P5] *“We get graded on doing, not being critiquers. That’s the professors’ job*”. Such reflections illustrate the perceived risks of dissent, as authority bias seemingly normalizes the performative identity.

It is possible evaluative practices reinforce this identity, privileging task completion over reflection. Report cards, for instance, rarely value contemplative efforts; instead, they emphasize adherence to structured outputs, often framing noncompliance as a lack of focus or effort (Daff et al., 2024). In part, this risks creating a generation of learners adept at execution but ill-prepared for the nuanced problem-solving required in professional and societal contexts (Sudirman et al., 2024), perpetuating a cycle of unexamined compliance that extends beyond the classroom into broader institutional and organizational settings (Schürmann et al., 2025).

*a.*  *Importance and Relevance of the Findings*

These educational paradigms could carry meaningful implications in an era characterized by algorithmic decision-making, fabricated online content, and information saturation. Students conditioned to prioritize action before critical inquiry are particularly vulnerable in navigating the complexities of digital environments. Ng et al.’s (2023) research on digital literacy highlights the crucial importance of teaching learners to assess the credibility of information, a skill that can be potentially undermined by pedagogical structures that reward reflexive task completion. As one student aptly captured, [B-P6] “*It’s not just about assignments. If we don’t stop to question, it’s easy to accept everything we are told or read*.”

In the context of an artificial intelligence (AI) era, where misinformation can proliferate alongside credible content (Sun et al., 2024), the ability to engage in thoughtful, problem-oriented inquiry becomes essential. Action bias, particularly when reinforced by educational and social norms, may limit individuals’ capacity to approach complex problems with the critical rigor required for accurate analysis and ethical decision-making. The behavior observed in this study thus motivates exploration of educational models that value the *process of learning* (Lachman, 1997), where the focus is placed not on immediate solutions but on the intellectual rigor necessary to question assumptions and validate the relevance of problems.

*b.*  *Propositions for Education*

To prepare learners for the complexities of a rapidly evolving world, our analysis supports trialing curricula that balance decisiveness with reflection and that value not only outcomes but the process of learning. This need is heightened by the growing accessibility of generative AI, which can effortlessly produce surface-level polished work (Sun et al., 2024; Elsayed, 2024), raising critical questions about what constitutes evidence of achieving learning outcomes.

Table 3 contrasts two distinct approaches to demonstrating learning outcomes: *evidence of achieved learning outcomes* and *evidence of the process of learning outcomes*. Traditional methods emphasize static, summative outputs which can demonstrate knowledge mastery at a fixed point (e.g., Gijbels et al., 2005; Nicol & Macfarlane-Dick, 2006) yet can devalue the messy and iterative process of learning. Recent studies reinforce this by showing that embedding reflective practices fosters deeper engagement and critical thinking, which traditional summative approaches often overlook (Sudirman et al., 2024; Schürmann et al., 2025). For instance, in Table 4 Learning Outcome (LO) 1: *Describe the types of insect life cycles*, traditional assessment evidence might include a multiple-choice test, case study, or essay, providing a snapshot of factual recall and organization. However, if a polished, AI-assisted essay no longer serves as a definitive, authentic measure, we posit that educational practices must pivot toward capturing the *learning process itself* as evidence. This could include artifacts such as brainstorming notes, iterative drafts, or demonstrations of experiential learning, such as *A Day in the Life of* documentaries, recordings of collaborative peer-led discussions, live negotiations, or debates on contentious topics. Together, these approaches shift the emphasis from passive reproduction of knowledge to active, relevant learning that mirrors the complexities of real-world problem-solving.

However, reframing education will likely challenge entrenched, old ways of thinking. Therefore, achieving this vision requires sustained collaboration among educators, policymakers, and institutional leaders to design learning environments that value depth over speed, cognitive exploration over compliance, and intellectual inquiry over superficial outcomes.

## 5. Limitations

This study provides a valuable exploration of action bias within higher education, yet its limitations reflect both the chosen methodology and the complexities inherent in studying cognitive phenomena in educational settings. One limitation arises from the controlled design of the workshops. While this approach aligns with experiential learning theories (Kolb, 2014), the artificiality of the workshop environment may have influenced participants’ natural problem-solving behaviors. The role of the facilitator presents another potential source of bias. The lead author facilitated all sessions, and this may have introduced authority-related demand characteristics. Although standardized prompts and structured reflection were employed to minimize this effect, the potential influence of facilitator presence cannot be entirely excluded. A further consideration is the absence of manipulation checks for psychological safety and collaboration climate. Such measures could have provided additional assurance that the workshops were experienced as supportive environments and that contributions were not unduly shaped by peer or facilitator pressures.

Although supplementary workshops were conducted at each educational level to check for consistency, only one group per level was available. This design provided useful exploratory insights but remains insufficient to separate level-based differences from potential group-dynamics effects. The analytic approach was inductive-abductive and did not involve a priori hypotheses, meaning that the findings are interpretive and should be regarded as context-bound rather than confirmatory.

Beyond these design considerations, this study examined participants’ reflective sensemaking only immediately after the task and did not assess longer-term impacts. Reflection has been shown to play a critical role in mitigating cognitive biases (Schön, 2017), suggesting that longitudinal studies examining the persistence or attenuation of action bias after targeted interventions could provide valuable insights into effective pedagogical strategies. Finally, this study’s scope is constrained by the absence of triangulated data sources, such as diaries or observational notes, and by reliance on an English-only literature base that likely reflects the WEIRD sample bias noted earlier (Begeny et al., 2018; van de Vijver, 2013). Future research could integrate additional qualitative and quantitative streams to deepen understanding and refine context-sensitive frameworks for educational reform and cognitive development. Addressing these limitations will require sustained, multi-site investigations to test whether reflective prompts, metacognitive scaffolds, and inquiry-driven pedagogy can attenuate action bias across contexts and over time.

## 6. Future Research Directions

These considerations point to several critical avenues for exploring the relationship between action bias and educational practice, offering initial insights into how cognitive tendencies are shaped by institutional and cultural norms. Yet the complexity of this phenomenon calls for sustained inquiry to address gaps that this research has only begun to surface.

One important direction for future research lies in tracing the developmental trajectory of action bias. While this study provides a snapshot of its manifestation in higher education, longitudinal research could illuminate how cognitive tendencies evolve over time, from early schooling to professional life. This would allow scholars to identify key developmental inflection points where targeted pedagogical interventions could disrupt uncritical patterns and cultivate habits of reflective inquiry. Such work not only enhances theoretical models but also informs reforms aimed at fostering intellectual resilience and critical autonomy across the learning lifespan.

The intersection of action bias with broader systemic and cultural dynamics also presents fertile ground for interdisciplinary exploration. Combining perspectives from cognitive psychology, educational theory, and institutional sociology could reveal how organizational norms and pedagogical practices reinforce or mitigate bias. Comparative studies across educational systems would further clarify how cultural and structural variables shape learners’ approaches to problem-solving, and how institutions can better balance the demands of efficiency with the need for intellectual depth and cognitive flexibility.

The accelerating integration of generative AI in education adds new complexity. While this study gestures toward the risks of surface-level outputs, future research must examine how these technologies influence cognitive processes and learning behaviors. Do they reinforce impulsive problem-solving, or could they be leveraged to deepen engagement with complex, ambiguous tasks? Understanding these dynamics will be essential to developing tools that support, rather than replace, thoughtful learning.

Finally, the reconceptualization of assessment models warrants rigorous attention. Traditional frameworks that prioritize polished outcomes (e.g., Gijbels et al., 2005; Nicol & Macfarlane-Dick, 2006) risk perpetuating performative learning. Future research could explore alternative paradigms that foreground the processes of learning rather than its products. Collaborative projects, experiential learning tasks, and iterative problem-solving activities offer promising directions for redefining educational success. However, robust empirical evaluations are needed to determine how these models can effectively capture the multifaceted nature of intellectual engagement and critical inquiry, particularly in scalable and diverse educational settings.

## 7. Conclusions

This exploratory study provides preliminary evidence that action bias can emerge in educational contexts, suggesting that ingrained tendencies to prioritize immediate action over reflective inquiry can undermine the intellectual development and critical thinking that education seeks to achieve. By dissecting the intricate interplay between cognitive heuristics, institutional norms, and cultural conditioning, this research offers a compelling critique of traditional pedagogical practices (e.g., Gijbels et al., 2005; Nicol & Macfarlane-Dick, 2006). It reveals how these practices, often unwittingly, valorize output-driven behaviors at the expense of the metacognitive skills required for navigating complex, real-world challenges.

The findings suggest a need to reconsider education’s fundamental purpose. If education aims to cultivate not merely repositories of knowledge but adaptable, innovative thinkers, then it should challenge its culture of visible productivity. Instead, it should embrace intellectual depth, where the act of questioning is as celebrated as the act of answering. This paradigm shift necessitates a deliberate effort to dismantle systemic reinforcements of authority bias and performative academic identities, replacing them with environments that foster cognitive exploration and critical autonomy.

Moreover, this study situates action bias as a broader societal issue, reflecting the pressures of an age dominated by immediacy, algorithmic decision-making, and performative metrics of success. The tendency to equate decisiveness with competence, observed across multiple domains, is magnified within educational systems that reward conformity and compliance over intellectual risk-taking. Such dynamics perpetuate a cycle where students are conditioned to prioritize action without questioning, a behavior that extends into professional and societal spheres, exacerbating the challenges of navigating an increasingly complex world.

The implications of this research are far-reaching, calling for a reconceptualization of education as a space of thoughtful engagement over hurried output, aligning with global innovation-knowledge goals (Lytras et al., 2022). This transformation requires systemic change: curricula must prioritize inquiry-driven learning, assessments must value processes over products, and educators must model the intellectual humility and curiosity they seek to inspire. Additionally, the integration of emergent technologies, including AI, must be approached with caution, ensuring these tools enhance rather than erode reflective capacities.

Ultimately, the significance of this study lies in its call to action for a reimagining of education’s role in fostering intellectual resilience and depth. As we confront the uncertainties of an evolving global landscape, the capacity to think critically, question assumptions, and deliberate deeply will define our collective ability to innovate, adapt, and thrive. This study thus serves as both a critique and a blueprint, urging educators, policymakers, and scholars to embrace a more reflective, inquiry-driven paradigm that equips learners to engage meaningfully with the complexities of the future.

## Figures and Tables

**Table 1:** Inclusion and exclusion criteria.

Stage	Inclusion Criteria	Exclusion Criteria
**Title/Abstract Screening**	Peer-reviewed; English; relevant to action bias	Non-English; tangential references
**Full-text Screening**	Empirical or theoretical link to action bias in decision/learning	Climate-specific “single action bias”; tangential
**Full-text Screening**	Accessible full text	Inaccessible full text
**Duplicate Check**	Unique records	Duplicate records

**Table 2:** Literature Search.

Keywords	Identification	Screening	Included/Excluded
“Action Bias”	Database Scopus *n* = 95	Based on abstract and title, English language *n* = 26	Based on availability and relevance *n* = 10; Excluded—single action bias
“Action Bias AND Education”	Database Google Scholar *n* = 28	Based on abstract and title, English language *n* = 28	Based on availability and relevance *n* = 9; Excluded—2 duplicates
“Action Bias AND Innovation”	Database Google Scholar *n* = 15	Based on abstract and title, English language *n* = 15	Based on availability and relevance *n* = 3; Excluded—2 duplicates

**Table 3:** Participants’ Demographics.

Demographics	Group A	Group B	Group C
**Ethnicity (all grew up in NZ)**	5 × NZ Europeans 1 × Pacifica person	4 × NZ European 1 × Pakistani 1 × Middle Eastern	3 × NZ European 1 × East Asia 1 × Afghanistan 1 × South Asia
**Age Range**	18–19 years old	19–21 years old	23–27 years old
**Gender Identity**	3 × females 3 × males	4 × females 2 × males	4 × females 2 × males

**Table 4:** Example of Traditional Versus Modern Assessments.

Learning Outcome (LO)	Traditional Evidence of Learning Outcomes Achieved	Evidence of the Process of Learning Outcomes
**1. Describe the types of insect life cycles**	- A completed essay detailing insect life cycles. - Multiple-choice test results on lifecycle terminology.	- Annotated visual diagrams with iterative improvements. - Peer-reviewed storyboard explaining life cycles. - Journal reflections on conceptual understanding.
**2. Explain how the stages of the life cycle can be manipulated to minimize harmful effects and maximize beneficial effects**	- Case study report detailing manipulation techniques. - Written test on strategies for lifecycle management.	- Group simulation exercises with reflections on decision-making. - Drafts of a pest control plan, with annotated changes based on feedback. - Role-play logs documenting adaptive problem-solving.
**Focus**	Final demonstration of mastery through static, polished outputs.	Documentation of growth, critical thinking, and collaboration throughout the learning process.
**Assessment Purpose**	Validate content knowledge and factual recall.	Capture dynamic engagement, creativity, and iterative refinement of ideas.
**Evidence Collected**	- Essays, tests, and case studies.	- Drafts, reflections, collaborative recordings, annotated artifacts, and multimedia presentations.
**Advantages**	- Standardized, easily comparable metrics. - Efficient for summative evaluations.	- Reflects authentic learning. - Encourages deeper intellectual engagement and transferable skill development. - Resilient to AI misuse.
**Key References**	- Biggs (1996)	- Kolb (2014) - Lachman (1997)

## Data Availability

Data is unavailable due to privacy or ethical restrictions.

## References

[B1-behavsci-15-01372] Abrami P. C., Bernard R. M., Borokhovski E., Waddington D. I., Wade C. A., Persson T. (2015). Strategies for teaching students to think critically: A meta-analysis. Review of Educational Research.

[B2-behavsci-15-01372] Ariely D. (2008). Predictably irrational: The hidden forces that shape our decisions.

[B3-behavsci-15-01372] Bailin S., Case R., Coombs J. R., Daniels L. B. (1999). Conceptualizing critical thinking. Journal of Curriculum Studies.

[B4-behavsci-15-01372] Bar-Eli M., Azar O. H., Ritov I., Keidar-Levin Y., Schein G. (2007). Action bias among elite soccer goalkeepers: The case of penalty kicks. Journal of Economic Psychology.

[B5-behavsci-15-01372] Begeny J. C., Levy R. A., Hida R., Norwalk K., Field S., Suzuki H., Soriano-Ferrer M., Scheunemann A., Guerrant M., Clinton A., Aguirre Burneo C. (2018). Geographically representative scholarship and internationalization in school and educational psychology: A bibliometric analysis of eight journals from 2002–2016. Journal of School Psychology.

[B6-behavsci-15-01372] Begeny J. C., van Schalkwyk G. J., Kim E. K., Datu J. A., Hida R. M., Wang J., Grazioso M. P., Floyd R. G., Eckert T. L. (2021). Engaging internationally to produce scholarship in school and educational psychology: A critical perspective. Handbook of university and professional careers in school psychology.

[B7-behavsci-15-01372] Biggs J. (1996). Enhancing teaching through constructive alignment. Higher Education.

[B8-behavsci-15-01372] Borowa K., de Almeida R. R., Wiese M. (2025). Debiasing architectural decision-making: An experiment with students and practitioners. 2025 IEEE 22nd International Conference on Software Architecture (ICSA).

[B9-behavsci-15-01372] Boud D., Cohen R., Sampson J. (2014). Peer learning in higher education: Learning from and with each other.

[B10-behavsci-15-01372] Brookfield S. (2013). Teaching for critical thinking. International Journal of Adult Vocational Education and Technology.

[B11-behavsci-15-01372] Campbell A. V., Wang Y., Inzlicht M. (2024). Experimental evidence that exerting effort increases meaning.

[B12-behavsci-15-01372] Carland M. A., Thura D., Cisek P. (2019). The urge to decide and act: Implications for brain function and dysfunction. The Neuroscientist.

[B13-behavsci-15-01372] Charmaz K. (2006). Constructing grounded theory: A practical guide through qualitative analysis.

[B14-behavsci-15-01372] Chipeta E. M., Venter R., Kruse P. (2022). Measuring the role of reductive bias in social enterprise formation: Development and validation of a social entrepreneurial intention bias scale. Journal of Social Entrepreneurship.

[B15-behavsci-15-01372] Cioffi J. (2025). Action for cognitive biases in clinical decision-making. Academia Medicine.

[B16-behavsci-15-01372] Colas J. T., O’Doherty J. P., Grafton S. T. (2024). Active reinforcement learning versus action bias and hysteresis: Control with a mixture of experts and nonexperts. PLoS Computational Biology.

[B17-behavsci-15-01372] Corbin J., Strauss A. (2014). Basics of qualitative research: Techniques and procedures for developing grounded theory.

[B18-behavsci-15-01372] Daenekindt S., Huisman J. (2020). Mapping the scattered field of research on higher education: A correlated topic model of 17,000 articles, 1991–2018. Higher Education.

[B19-behavsci-15-01372] Daff L., Tame C., Sands J. (2024). A course design approach that encourages reflective practice habits. The International Journal of Management Education.

[B20-behavsci-15-01372] Decker D. J., Pomeranz E. F., Forstchen A. B., Riley S. J., Lederle P. E., Schiavone M. V., Baumer M. S., Smith C. A., Frohlich R. K., Benedict R. J., King R. (2022). Taking time to think: The tyranny of being “too busy” and the practice of wildlife management. Frontiers in Conservation Science.

[B21-behavsci-15-01372] Dykstra J., Met J., Backert N., Mattie R., Hough D. (2022). Action bias and the two most dangerous words in cybersecurity incident response: An argument for more measured incident response. IEEE Security & Privacy.

[B22-behavsci-15-01372] Elder L., Paul R. (2009). Critical thinking: Strategies for improving student learning, Part III. Journal of Developmental Education.

[B23-behavsci-15-01372] Elsayed H. (2024). The impact of hallucinated information in large language models on student learning outcomes: A critical examination of misinformation risks in AI-assisted education. Northern Reviews on Algorithmic Research, Theoretical Computation, and Complexity.

[B24-behavsci-15-01372] Ennis R. (2011). Critical thinking: Reflection and perspective Part II. Inquiry: Critical Thinking Across the Disciplines.

[B25-behavsci-15-01372] Forbes J. N., Farrar M. J. (1993). Children’s initial assumptions about the meaning of novel motion verbs: Biased and conservative?. Cognitive Development.

[B26-behavsci-15-01372] Foucault M. (2012). Discipline and punish: The birth of the prison.

[B27-behavsci-15-01372] Freire P. (2020). Pedagogy of the oppressed.

[B28-behavsci-15-01372] Friesen N., Osguthorpe R. (2018). Tact and the pedagogical triangle: The authenticity of teachers in relation. Teaching and Teacher Education.

[B29-behavsci-15-01372] Fynes-Clinton E., Burgh G., Thornton S. (2024). Toward a self-correcting society: Deep reflective thinking as a theory of practice. Journal of Philosophy in Schools.

[B30-behavsci-15-01372] Gernsbacher M. A., Soicher R. N., Becker-Blease K. A. (2020). Four empirically based reasons not to administer time-limited tests. Translational Issues in Psychological Science.

[B31-behavsci-15-01372] Gijbels D., Dochy F., Van den Bossche P., Segers M. (2005). Effects of problem-based learning: A meta-analysis from the angle of assessment. Review of Educational Research.

[B32-behavsci-15-01372] Gioia D. A., Corley K. G., Hamilton A. L. (2013). Seeking qualitative rigor in inductive research: Notes on the Gioia methodology. Organizational Research Methods.

[B33-behavsci-15-01372] Guest G., Bunce A., Johnson L. (2006). How many interviews are enough? An experiment with data saturation and variability. Field Methods.

[B34-behavsci-15-01372] Gültekin D. G., Ali F., Demir G. (2024). Understanding and mitigating authority bias in business and beyond. Overcoming cognitive biases in strategic management and decision making.

[B35-behavsci-15-01372] Hallström J., Schönborn K. J. (2019). Models and modelling for authentic STEM education: Reinforcing the argument. International Journal of STEM Education.

[B36-behavsci-15-01372] Halpern D. F. (1998). Teaching critical thinking for transfer across domains: Dispositions, skills, structure training, and metacognitive monitoring. American Psychologist.

[B37-behavsci-15-01372] Halpern D. F. (2013). Thought and knowledge: An introduction to critical thinking.

[B38-behavsci-15-01372] Harmon-Jones E., Matis S., Angus D. J., Harmon-Jones C. (2024). Does effort increase or decrease reward valuation? Considerations from cognitive dissonance theory. Psychophysiology.

[B39-behavsci-15-01372] Hertwig R., Erev I. (2009). The description–experience gap in risky choice. Trends in Cognitive Sciences.

[B40-behavsci-15-01372] Iftekhar M. S., Pannell D. J. (2015). “Biases” in adaptive natural resource management. Conservation Letters.

[B41-behavsci-15-01372] Iserson K. V. (2024). Informed consent for artificial intelligence in emergency medicine: A practical guide. The American Journal of Emergency Medicine.

[B42-behavsci-15-01372] Kahneman D. (2011). Fast and slow thinking.

[B43-behavsci-15-01372] Kolb D. A. (2014). Experiential learning: Experience as the source of learning and development.

[B44-behavsci-15-01372] Lachman S. J. (1997). Learning is a process: Toward an improved definition of learning. The Journal of Psychology.

[B45-behavsci-15-01372] Lamiraud K., Vranceanu R. (2018). Group gender composition and economic decision-making: Evidence from the Kallystée business game. Journal of Economic Behavior & Organization.

[B46-behavsci-15-01372] Leibovitch Y. M., Beencke A., Ellerton P. J., Mcbrien C., Robinson-Taylor C. L., Brown D. J. (2025). Teachers’(evolving) beliefs about critical thinking education during professional learning: A multi-case study. Thinking Skills and Creativity.

[B47-behavsci-15-01372] Li X., Chen W., Alrasheedi M. (2023). Challenges of the collaborative innovation system in public higher education in the era of industry 4.0 using an integrated framework. Journal of Innovation & Knowledge.

[B48-behavsci-15-01372] Liu O. L., Frankel L., Roohr K. C. (2014). Assessing critical thinking in higher education: Current state and directions for next-generation assessment. ETS Research Report Series.

[B49-behavsci-15-01372] Lytras M. D., Serban A. C., Ruiz M. J. T., Ntanos S., Sarirete A. (2022). Translating knowledge into innovation capability: An exploratory study investigating the perceptions on distance learning in higher education during the COVID-19 pandemic-the case of Mexico. Journal of Innovation & Knowledge.

[B50-behavsci-15-01372] Milgram S. (1963). Behavioral study of obedience. Journal of Abnormal and Social Psychology.

[B51-behavsci-15-01372] Ng D. T. K., Ng R. C. W., Chu S. K. W. (2023). Engaging students in virtual tours to learn language and digital literacy. Journal of Computers in Education.

[B52-behavsci-15-01372] Nicol D. J., Macfarlane-Dick D. (2006). Formative assessment and self-regulated learning: A model and seven principles of good feedback practice. Studies in Higher Education.

[B53-behavsci-15-01372] Noel T., Erskine L. (2013). The silent story: Using computer-aided text analysis to predict entrepreneurial performance. The Journal of Entrepreneurship.

[B54-behavsci-15-01372] Okonofua J. A., Walton G. M., Eberhardt J. L. (2016). A vicious cycle: A social–psychological account of extreme racial disparities in school discipline. Perspectives on Psychological Science.

[B55-behavsci-15-01372] Ormerod T. C., MacGregor J. N., Chronicle E. P., Dewald A. D., Chu Y. (2013). Act first, think later: The presence and absence of inferential planning in problem solving. Memory & Cognition.

[B56-behavsci-15-01372] Patt A., Zeckhauser R. (2000). Action bias and environmental decisions. Journal of Risk and Uncertainty.

[B57-behavsci-15-01372] Paukku M., Välikangas L. (2021). Harried or myopic leadership: An undue bias for action. Debating bad leadership: Reasons and remedies.

[B58-behavsci-15-01372] Paz-Baruch N., Grovas G., Mevarech Z. R. (2025). The effects of meta-creative pedagogy on elementary school students’ creative thinking. Metacognition and Learning.

[B59-behavsci-15-01372] Philp-Clark C., Grieshaber S. (2024). Teacher critical reflection: What can be learned from quality research?. The Australian Educational Researcher.

[B60-behavsci-15-01372] Radics R., Dasmohapatra S., Kelley S. S. (2015). Systematic review of bioenergy perception studies. BioResources.

[B61-behavsci-15-01372] Reason J. (2009). The cognitive bases of predictable human error. Contemporary ergonomics 1984–2008: Selected papers and an overview of the ergonomics society annual conference.

[B62-behavsci-15-01372] Sandberg J., Tsoukas H. (2015). Making sense of the sensemaking perspective: Its constituents, limitations, and opportunities for further development. Journal of Organizational Behavior.

[B63-behavsci-15-01372] Schön D. A. (2017). The reflective practitioner: How professionals think in action.

[B64-behavsci-15-01372] Schürmann V., Bodemer D., Marquardt N. (2025). Exploring the use of regular reflections in student collaboration: A case study in higher education. Frontiers in Education.

[B65-behavsci-15-01372] Shavelson R. (2010). Measuring college learning responsibly: Accountability in a new era.

[B66-behavsci-15-01372] Shiller R. J. (2000). Irrational exuberance.

[B67-behavsci-15-01372] Shye S., Viale R. (2025). The cognitive basis for decision making under risk and uncertainty: Research programs & controversies. Frontiers in Psychology.

[B68-behavsci-15-01372] Sternberg R. J. (2002). Why smart people can be so stupid.

[B69-behavsci-15-01372] Sternberg R. J., Tardif T. (2009). The nature of creativity. The essential Sternberg: Essays on intelligence, psychology and education.

[B70-behavsci-15-01372] Sudirman A., Gemilang A. V., Kristanto T. M. A., Robiasih R. H., Hikmah I. A., Nugroho A. D., Karjono J. S., Lestari T., Widyarini T. L., Prastanti A. D., Susanto M. R. (2024). Reinforcing reflective practice through reflective writing in higher education: A systematic review. International Journal of Learning, Teaching and Educational Research.

[B71-behavsci-15-01372] Sun Y., Sheng D., Zhou Z., Wu Y. (2024). AI hallucination: Towards a comprehensive classification of distorted information in artificial intelligence-generated content. Humanities and Social Sciences Communications.

[B72-behavsci-15-01372] Sunstein C. R., Zeckhauser R. (2011). Overreaction to fearsome risks. Environmental and Resource Economics.

[B73-behavsci-15-01372] Thalmayer A. G., Toscanelli C., Arnett J. J. (2021). The neglected 95% revisited: Is American psychology becoming less American?. American Psychologist.

[B74-behavsci-15-01372] Tsui L. (2002). Fostering critical thinking through effective pedagogy: Evidence from four institutional case studies. The Journal of Higher Education.

[B76-behavsci-15-01372] van der Toorn J., Tyler T. R., Jost J. T. (2011). More than fair: Outcome dependence, system justification, and the perceived legitimacy of authority figures. Journal of Experimental Social Psychology.

[B75-behavsci-15-01372] van de Vijver F. J. (2013). Contributions of internationalization to psychology: Toward a global and inclusive discipline. American Psychologist.

[B77-behavsci-15-01372] Xu L., Barrow M. (2024). ‘Playing the same game differently’: Constituting academic identities in four disciplines. Higher Education.

[B78-behavsci-15-01372] Yulin N., Danso S. D. (2025). Assessing pedagogical readiness for digital innovation: A mixed-methods study. arXiv.

